# Finite Element Simulation of Ultrasonic Scattering by Rough Flaws with Multi-Scale Distortions

**DOI:** 10.3390/ma15238633

**Published:** 2022-12-03

**Authors:** Zheng Wang, Zhanhong Zeng, Yongfeng Song, Xiongbing Li

**Affiliations:** 1 Beijing Institute of Aeronautical Materials, Beijing 100095, China; 2 School of Traffic and Transportation Engineering, Central South University, Changsha 410075, China

**Keywords:** ultrasonic finite element simulation, multi-scale ripple distortions, flaw scattering, rough flaw, nickel-based bars

## Abstract

The roughness of a flaw’s surface significantly affects the scattering behavior of ultrasonic waves. It is vital to understand the impact of roughness on flaw echoes, especially when performing ultrasonic nondestructive inspection on safety-critical components. However, the current approach for creating rough flaw models fails to reconstruct complicated cracks with secondary cracks. Here, a multi-scale distortion method is developed to generate a rough flaw by using an optical microscope image of a real flaw. The finite element (FE) is then implemented to simulate the near-surface rough flaws in nickel-based bars, which are detected by an offsetting immersion transducer with mode-converted transverse waves. Numerical results show that the randomness and complexity of flaw echoes from rough flaws are exceptionally high. The gap between the maximum and minimum normalized amplitude values of flaw echoes from a rough crack with secondary cracks can reach 7.125 dB. Meanwhile, the maximum time of flight (TOF) is almost twice as large as the minimum TOF. Therefore, the present method can generate effective rough flaw models in terms of macroscopic rough geometry and microscopic rough surface. Moreover, the impact of the rough flaw surface on the flaw echoes goes beyond amplitude changes and may make flaw location challenging.

## 1. Introduction

Ultrasonic nondestructive inspection is dependent on the scattering of elastic waves by flaws. Hence, the scattering behavior of flaws must be investigated to improve the inspection scheme. Schmerr et al. [1] highlighted that the flaw scattering amplitude is strongly connected to the flaw echo signal and plays a crucial role in flaw sizing. However, real flaws are generally not standard reflectors with regular shapes (e.g., notch, void, and column), and changes in macroscopic rough geometry will alter the fundamental characteristics of flaw scattering. Meanwhile, real flaws do not have an ideally smooth surface. In addition, scattering caused by microscopic rough surfaces can significantly degrade signal amplitude [2,3,4] and should not be ignored. Thus, the impact of the surface roughness of flaws on ultrasonic inspection and quantitative evaluation should be analyzed at multiple scales: from macro to micro.

Most studies on elastic wave scattering from a rough surface rely on computer simulations, with rough surface reconstruction serving as the most important part. However, the development of rough surface reconstruction is currently primarily focused on microscopic rough surfaces. For example, Ogilvy [5] used the moving average process, the Gaussian height distribution, and the Gaussian surface correlation function (SCF) to reconstruct the rough surface. Monte Carlo simulations were then performed to obtain the statistical features of ultrasonic scattering. However, the point number of moving average cannot be excessively large or excessively small. Zhang et al. [6] and Pettit et al. [7] also employed the Gaussian SCF to reconstruct a one-dimensional (1D) rough surface, where the convolution operation for the Gaussian white noise was emphasized. Monte Carlo finite element (FE) simulations were conducted using the 1D rough surface. Their goal was to verify the analytical models using the Kirchhoff approximation. However, in reality, there is a significant difference between a 1D rough surface and three-dimensional (3D) rough surface. Jarvis and Cegla [8,9] also reconstructed a 1D rough surface with a SCF-based method, but the scattering events were simulated by the distributed point source method (DPSM).

Shi et al. [10,11] used the SCF-based method to reconstruct a two-dimensional (2D) rough surface. They utilized the 3D FE boundary integration approach to analyze the rough-surface scattering. Recently, Choi and Shi et al. [12] enhanced the SCF-based method by moving average and auto-regressive (AR) methods. They found that the AR surfaces showed the best agreement with the real rough surfaces. Even though the SCF-based approach has made significant progress in numerical simulations for microscopic surface roughness, it cannot simultaneously account for the macroscopic rough geometry in surface modeling. In addition, the Weierstrass-Mandelbrot (W-M) function [13] can be used to reconstruct a fractal-regular rough surface, but the W-M function cannot be used to create a complex rough surface with a primary profile. Obviously, the existing approaches are inadequate for simulating a primary crack with secondary cracks, because it is difficult to extract and reset the profile of a crack surface. Consequently, this work aims to develop a universal method for surface modeling at multiple scales, which will lead to a more realistic ultrasonic FE simulation.

In this work, we introduce an image distortion technique [14,15] into the ultrasonic FE simulation. This is a random perturbation approach that can produce rough-surfaced flaws with multi-scale distortions, according to an optical microscope (OM) image of a real flaw. The FE simulations were placed in a scenario where an offsetting immersion transducer used mode-converted transverse waves to detect near-surface flaws in nickel-based alloy bars. Finally, the simulation results of the flaw echoes from secondary cracks, sub-surface pores, and sub-surface inclusion were statistically analyzed and the effects of flaw scattering behavior with various rough surfaces were discovered.

## 2. Materials and Methods

### 2.1. Materials

Nickel-based alloy bars are widely used in the aerospace industry, due to their superior high-temperature strength, oxidation resistance, corrosion resistance, and fatigue resistance [16]. However, flaws are inevitably produced in nickel-based bars during the manufacturing process, which affects their performance and service safety. For instance, cracks, pores, and inclusions are frequently observed on the surface and subsurface of these bars [17,18,19,20]. Over the history of our extensive inspection experience, we have seldom found smooth flaws in nickel-based alloy bars, and the accurate ultrasonic estimation of flaw size is complicated by the presence of rough surfaces. Thus, nickel-based alloy bars are selected for the ultrasonic FE simulation in this work. Table 1 shows the input material properties that are associated with the nickel-based alloy, and one common inclusion of this alloy.

### 2.2. Generating Rough Flaws with Multi-Scale Distortions

**Figure 1:**
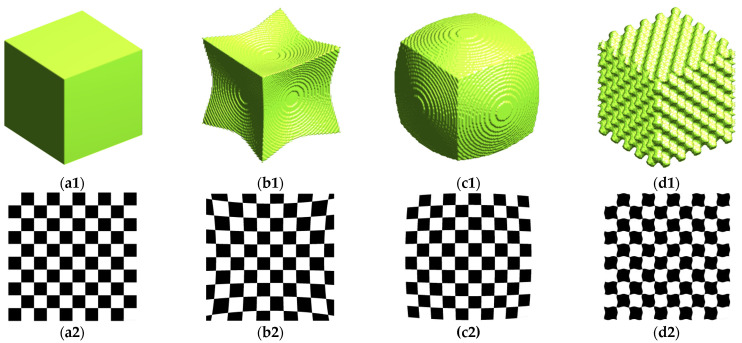
Schematic diagram of the distortions of a 3D cube and a 2D checkerboard: (**a1**) original image; (**b1**) pincushion distortion; (**c1**) barrel distortion; (**d1**) ripple distortion; (**a2**) original image; (**b2**) pincushion distortion; (**c2**) barrel distortion; (**d2**) ripple distortion.

Image distortion is defined as the aberrant or warped representation of an object by an optical imaging system. This phenomenon must be suppressed and corrected in the field of optical imaging. Figure 1 illustrates the pincushion, barrel, and ripple distortions of a 3D cube and a 2D checkerboard, where the pincushion distortion and barrel distortion phenomena originate from a lens’s misaligned edge-to-center magnification, and the ripple distortion phenomenon originates from the imaging difficulties caused by water-surface ripples. However, we suggest that the image distortion technique could be employed to artificially create rough flaws into nickel-based alloy bars. It is possible to generate distorted images at multiple scales by randomly perturbing the seed of a flaw image with different distortion parameters. This paves the way for an accurate simulation of the ultrasonic response characteristics of rough flaws, which will be described in the next section.

First, image distortion is essentially coordinate mapping, which can be achieved by converting the coordinates according to various mapping functions, and assigning the values under the original coordinates to the new coordinates, as follows:

(1) $$\begin{array}{c} x_{d}=x+\delta_{x}\left(x,y,z\right),\\ y_{d}=y+\delta_{y}\left(x,y,z\right),\\ z_{d}=z+\delta_{z}\left(x,y,z\right), \end{array}$$

where x, y and z are the original coordinates; $x_{d}$, $y_{d}$ and $z_{d}$ are the new coordinates after distortion; and $\delta_{x}$, $\delta_{y}$ and $\delta_{z}$ are distortions in three directions. The mathematical definition of ripple distortion, pincushion distortion, and barrel distortion can usually be expressed as [14]:

ripple distortion:

(2) $$\begin{array}{c} \delta_{x}=K\cdot \cos\left(\frac{2\pi }{Τ}y+\varphi \right)\cdot \cos\left(\frac{2\pi }{Τ}z+\varphi \right),\\ \delta_{y}=K\cdot \cos\left(\frac{2\pi }{Τ}z+\varphi \right)\cdot \cos\left(\frac{2\pi }{Τ}x+\varphi \right),\\ \delta_{z}=K\cdot \cos\left(\frac{2\pi }{Τ}x+\varphi \right)\cdot \cos\left(\frac{2\pi }{Τ}y+\varphi \right), \end{array}$$

pincushion distortion and barrel distortion:

(3) $$\begin{array}{c} \delta_{x}=\mathrm{sgn}x\cdot K\cdot \cos\left(\frac{2\pi }{Τ}\sqrt{y^{2}+z^{2}}+\varphi \right),\\ \delta_{y}=\mathrm{sgn}y\cdot K\cdot \cos\left(\frac{2\pi }{Τ}\sqrt{z^{2}+x^{2}}+\varphi \right),\\ \delta_{z}=\mathrm{sgn}z\cdot K\cdot \cos\left(\frac{2\pi }{Τ}\sqrt{x^{2}+y^{2}}+\varphi \right), \end{array}$$

where K, T and φ are the coefficients of image distortion; K is the distortion amplitude; T is the period term; and φ is the phase term. It should be noted that a positive K value results in pincushion distortion, while a negative K value results in barrel distortion. When the z coordinate is unavailable, the 3D distortion degrades to a 2D distortion (let z=0). It is difficult to count the flaw response under the independent identically distributed (IID) condition, because pincushion distortion and barrel distortion tend to cause significant changes in the flaw’s size. Therefore, we chose the ripple distortion technique for multi-scale perturbation of the seed of the flaw image.

**Figure 2:**
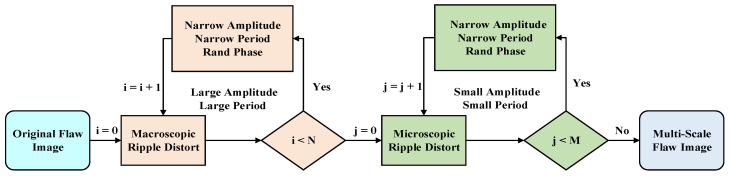
Flowchart of multi-scale distortion.

As shown in the flowchart in Figure 2, the original flaw image is preprocessed and progressively distorted according to specified distortion parameters. For macroscopic distortion, the random distortion amplitude is $K^{\left(i\right)}∼U\left(K_{1}^{\left(i\right)},K_{2}^{\left(i\right)}\right)$, the random period is $T^{\left(i\right)}∼U\left(T_{1}^{\left(i\right)},T_{2}^{\left(i\right)}\right)$ and the random phase is $\varphi ∼U\left(0,2\pi \right)$, where $U\left(\cdot \right)$ denotes the uniform distribution; $K_{1}^{\left(i\right)}<K_{2}^{\left(i\right)}$ and; $K_{1,2}^{\left(i\right)}>K_{1,2}^{\left(i+1\right)}$ and $T_{1,2}^{\left(i\right)}>T_{1,2}^{\left(i+1\right)}$; i=0,1,⋯,N−1; and N is the number of macroscopic distortions. For microscopic distortion, the random distortion amplitude is $K^{\left(j\right)}∼U\left(K_{3}^{\left(j\right)},K_{4}^{\left(j\right)}\right)$, the random period is $T^{\left(j\right)}∼U\left(T_{3}^{\left(j\right)},T_{4}^{\left(j\right)}\right)$ and the random phase is also $\varphi ∼U\left(0,2\pi \right)$, where $K_{3}^{\left(j\right)}<K_{4}^{\left(j\right)}$ and $T_{3}^{\left(j\right)}<T_{4}^{\left(j\right)}$; $K_{3,4}^{\left(j\right)}>K_{3,4}^{\left(j+1\right)}$ and $T_{3,4}^{\left(j\right)}>T_{3,4}^{\left(j+1\right)}$; j=0,1,⋯,M−1; and M is the number of microscopic distortions. $K_{1,2}$, $K_{3,4}$, $T_{1,2}$, and $T_{3,4}$ are the hyper-parameters of the uniform distributions. Notice that $K_{3,4}\ll K_{1,2}$ and $T_{3,4}\ll T_{1,2}$ reflect the division between macroscopic and microscopic scales. Continuous distortions can be achieved by nested calculations of Equations (1) and (2). Considering Equation (2) as part of the Fourier series, we may use the idea of spatial Fourier transform to help understand our method. In the spatial Fourier transform, the convolution calculation and transfer function can change the input image. Here, the amplitude, period, and phase parameters of the ripple distortion can be related to the module, wave number, and direction of the wave vector of the transfer function, respectively. Thus, any image transformation might be achieved by combining the appropriate parameters of the ripple distortion. In this work, we have randomly chosen a few discrete parameter combinations as samples. However, it is not necessary to always use uniform distribution; one may obtain the desired rough surface reconstruction by carefully designing the parameters combination for ripple distortion based on experimental observations in the future. Finally, repeating this process Q times results in Q flaws with various degrees of roughness.

**Figure 3:**
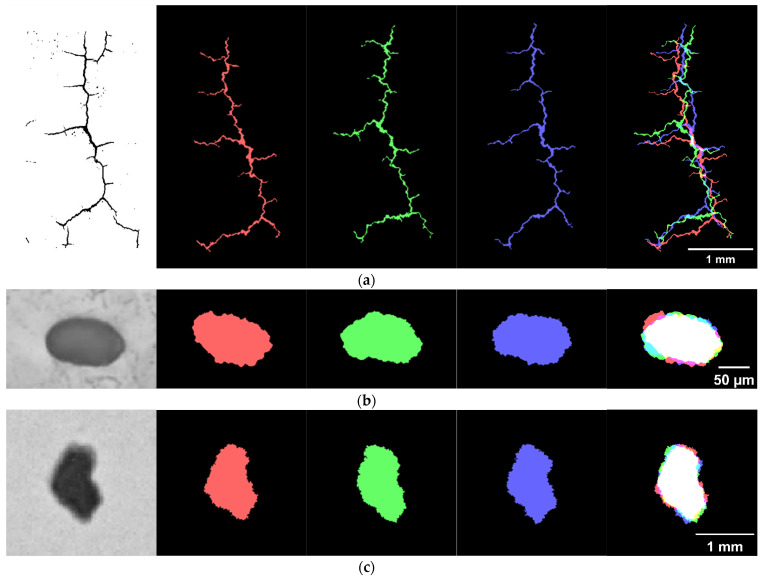
Distorted image: (**a**) a crack; (**b**) a pore; (**c**) an inclusion. The similarities and differences between three typical repetitions are highlighted using pseudo-color imaging, where the shared areas are in white.

Figure 3 shows three samples of seeds and the resulting multi-scale ripple distortion after multiple random perturbations. Let Q=100, and three typical rough flaws generated within 100 repetitions are exhibited for each seed. Figure 3a depicts a primary crack with several secondary cracks, which has seldom been discussed in ultrasonic simulation. This crack is equivalently ~0.02 mm wide and ~3 mm deep in the original OM images. Figure 3b,c demonstrate the multi-scale distortion results of a pore and a non-metallic inclusion, whose equivalent diameters in the original OM images are 0.1 mm and 1 mm, respectively. As observed in the pseudo-color images of Figure 3, flaws distorted by random ripples are notably more different in macroscopic rough geometry and microscopic rough surfaces than the seeds of the original flaw images.

Additionally, based on ISO 21920-2:2021, the arithmetic mean roughness Ra can be expressed as:

(4) $$Ra=\frac{1}{\ell }\int_{0}^{\ell }\left|Z\left(x\right)\right|dx$$

where Z and ℓ are the height and length of the roughness profile. To produce the roughness profile, the flaw’s primary profile or waviness profile must be eliminated. Thus, the edge extraction, moving average, and skeletonization methods were employed to calculate the primary profile, such that the roughness profile can be obtained by deviating from the primary profile in the normal direction. Consequently, the surface roughness Ra of 100 cracks, pores, and inclusions are: 10.85 ± 2.85 μm, 0.60 ± 0.37 μm, and 9.05 ± 3.15 μm, respectively. The roughness of the pore is one order of magnitude smaller than that of the other two flaws because the size of the pore is also one order of magnitude smaller.

### 2.3. Ultrasonic Finite Element Method

The ultrasonic inspection of nickel-based bars with a mode-converted transverse wave is simulated and analyzed with the discontinuous Galerkin time-domain (DGTD) method, which solves the sound field and pulse-echo signals from a focal transducer. Here, Q = 100 is the total number of simulation repetitions for each case. In each successive simulation, the rough defect model is altered by employing the multi-scale distortion technique. In addition, the attenuation coefficient of the nickel-based bar is considered to predict the flaw scattering amplitude with high accuracy. This simulation is built in COMSOL^®^ and MATLAB^®^ using an Intel CPU i9-10900K (10 cores, 5 GHz). The simulations will be verified by comparing them to the experimental results in Section 3.

#### 2.3.1. FE Simulation Setup

**Figure 4:**
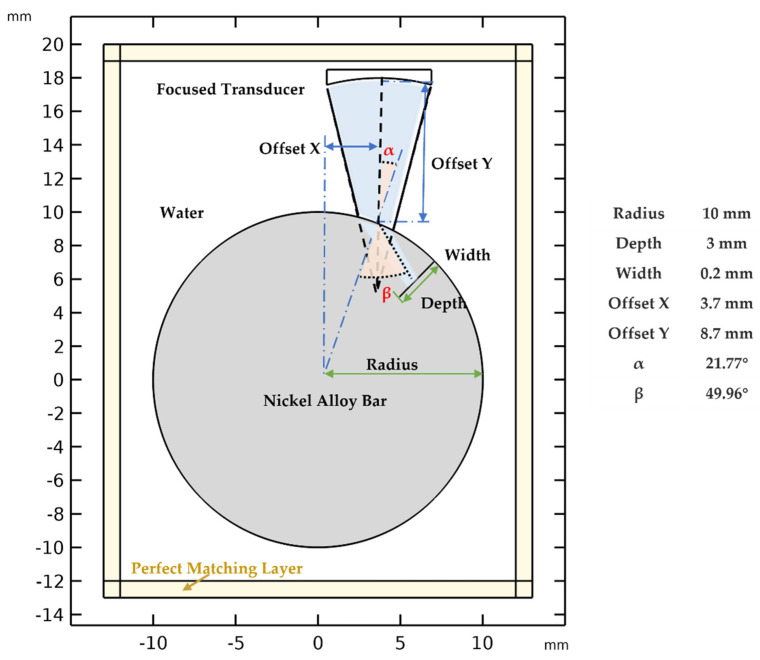
Setup for ultrasonic inspection of nickel-based bars with a mode-converted transverse wave.

Figure 4 shows the FE simulation setup for the ultrasonic inspection of nickel-based bars with a mode-converted transverse wave, which was simplified to a 2D model under the assumption of plane strain. The focal transducer was simulated using a concave circular arc, with an element radius of 3.175 mm, a focal length of 12.7 mm, and a water path of 8.7 mm. The radius of the nickel-based bar was set to 10 mm. According to Table 1, the corresponding longitudinal wave velocity of the nickel-based bar was 5640 m/s, its transverse wave velocity was 3067 m/s, and the longitudinal wave velocity in water was 1486 m/s. Based on Snell’s law, the first critical angle of the fluid/solid interface was 15.28°, and the second critical angle was 28.98°. To regulate the refraction angle to 45°, the incidence angle should be 21.77°. Hence, the transducer’s offset distance was adjusted to 3.7 mm. As a baseline for further simulations of rough flaws, an ideal open crack or notch was analyzed at first. The width of the notch was 0.2 mm and its depth was 3 mm, according to the real crack in Figure 3a. A perfectly matched layer of sound absorption is applied to reduce sidewall interference and boundary reflections. Considering that we must perform a large number of repeated simulations for a class of flaws, the center frequency of the transducer was set to 2.5 MHz to obtain simulation results within a certain computational time.

**Figure 5:**
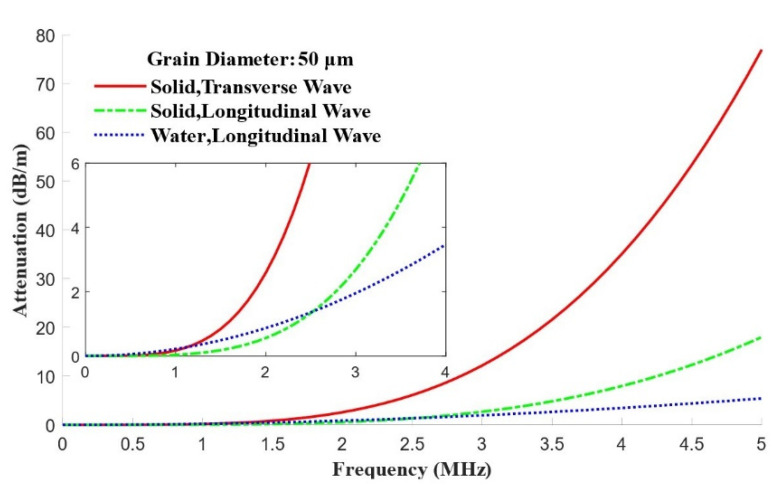
Attenuation coefficients in the frequency domain.

To make the simulation more realistic, material attenuation is added to COMSOL’s solid mechanics and acoustics modules to quantify the attenuation in solids and in water; their values are given in Figure 5. The frequency-dependent absorbing attenuation coefficient in water is $\alpha_{water}\left[\mathrm{dB}/m\right]=0.22f^{2}\left[\mathrm{MHz}\right]$, which is 1.36 dB/m at 2.5 MHz. The longitudinal and transverse wave attenuation coefficients in solids can be obtained from Weaver’s scattering attenuation model [23]. If the average grain diameter of the nickel-based bar is 50 µm, then the scattering attenuation coefficients of the longitudinal and transverse waves are 1.33 and 6.10 dB/m, respectively.

**Figure 6:**
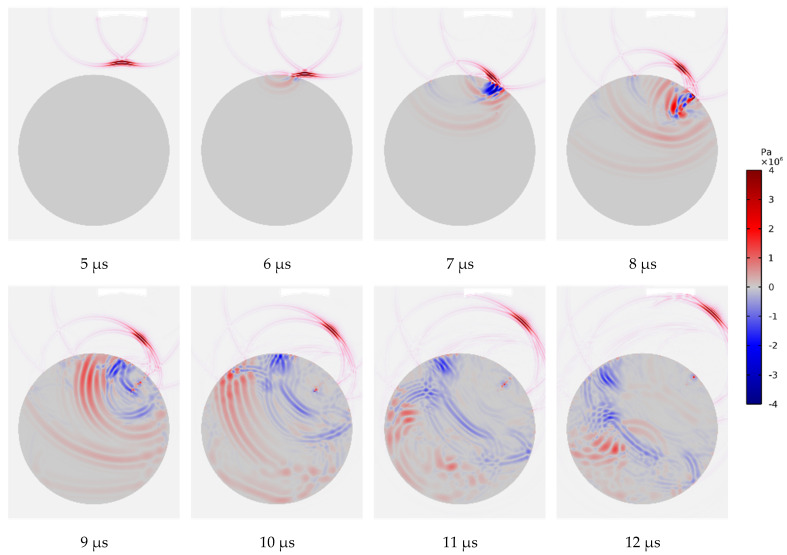
Snapshots of wave field for a smooth-surface notch.

Figure 6 gives the typical FEM results of flaw scattering from a smooth notch in the nickel-based bar. From the snapshots, we can see how the incident wave impacts the bar and converts into a transverse wave. Then, as the refracted wave travels to the smooth notch, the flaw scatters both the longitudinal and transverse wave components. Subsequently, the flaw scattering waves return to the solid/fluid interface. Finally, the flaw echo is received by the focal transducer. The imaging of snapshots is governed by the second stress invariant, with red representing the longitudinal wave component and blue representing the transverse wave component. The results that are presented in Figure 6 are consistent with the expectations.

#### 2.3.2. Model Verification

To demonstrate the reliability of the rough flaw simulations, ultrasonic experiments with a standard smooth notch were conducted to verify the simulations. A roller mechanism and a 2.5 MHz line focal transducer were utilized to perform a circular B-scan on a segment of nickel-based bar with a 10 mm radius and a 20 mm height, using a 1° angular scanning step. To be consistent with the simulation shown earlier, the offset distance was adjusted to 3.7 mm. This indicates that the incident angle is 21.77° and the corresponding refracted wave is 50°. In the radial direction, an open notch with a width of 0.2 mm and a depth of 3 mm was made into the nickel-based bar using low-speed wire cutting, and the roughness of the notch was 0.67 ± 0.18 μm.

**Figure 7:**
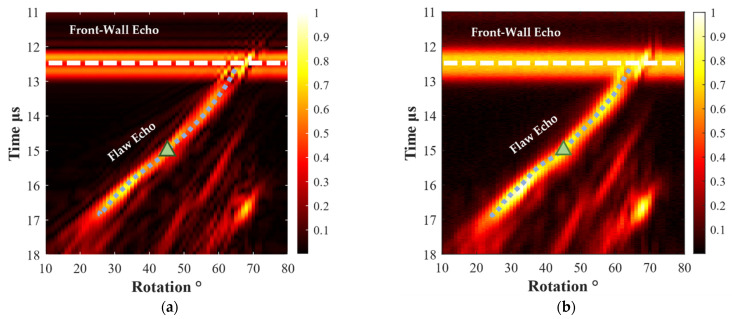
B scan image: (**a**) simulation; (**b**) experiment. The width of the notch was 0.2 mm.

**Figure 8:**
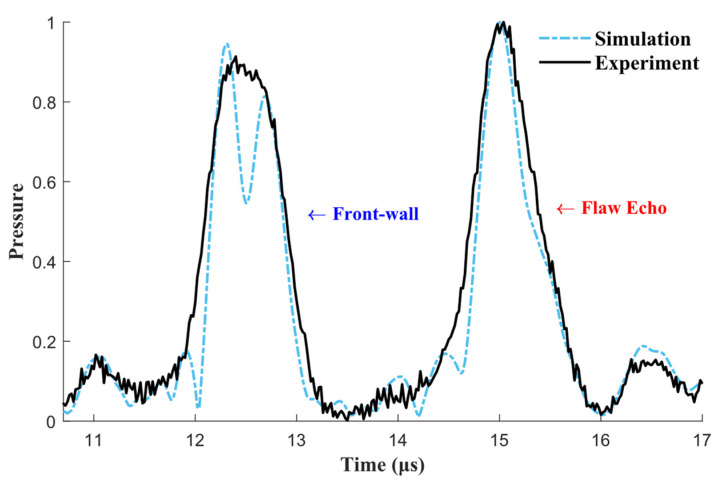
Pulse-echo signal when the notch is at 45° (normalized with the amplitude of each flaw echo).

The B-scan images from the simulation and the experiment are shown in Figure 7a and Figure 7b, respectively. There is substantial agreement between these two results. Because the refracted transverse wave is not always perpendicular to the notch, the time of flight (TOF) of the flaw echo is not a linear function of the scanning angle, as demonstrated in Figure 7 by the blue short-dot line. Here, TOF is the time difference between the front-wall echo and flaw echo on the time axis. To compare the simulated and measured TOFs of the notch, we recorded TOF values at a 45° scanning angle, which are marked by the upper triangles in Figure 7. In Figure 7a,b, the TOFs measure 2.52 and 2.64 μs, which indicates that their relative error is just 2.7%. Furthermore, Figure 8 shows the normalized flaw echoes corresponding to the markers. There is no obvious discrepancy between the simulated and measured flaw echoes. The discrepancy in the front-wall echoes might be due to the focus line not being on the surface, and the 2D simulation and 3D experiment at the non-focal point are different. Therefore, in subsequent simulations, a smooth notch will be replaced with various types of rough flaws to examine the influence of rough surfaces on flaw echoes.

## 3. Results and Discussion

In this section, ultrasonic inspections for various types of rough defects will be simulated based on the validated FE model (which was described earlier). The impact of the rough flaws on the flaw echoes will be explored using the crack, pore, and inclusion after multi-scale distortion, as shown in Figure 3. The sizes of all three flaws have previously been mentioned. However, the starting point of each rough crack lies on the bar’s surface, and the line between the starting point and centroid point of each rough crack passes through the center of the bar, while the pores and inclusions were positioned 1 mm beneath the surface. The other FE simulation setup is identical.

**Figure 9:**
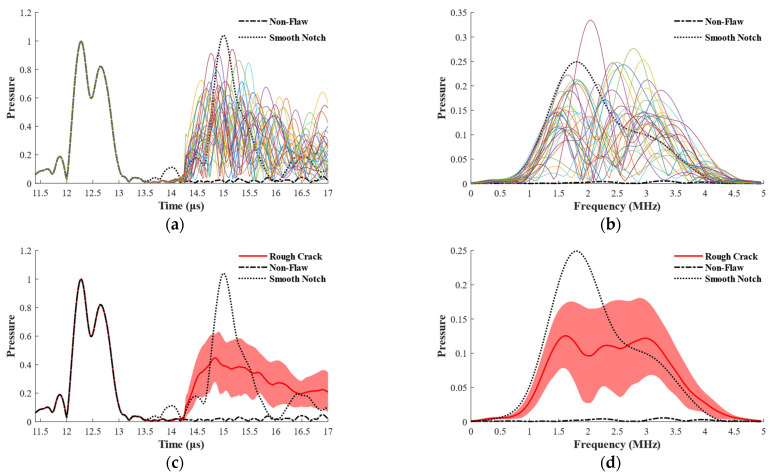
The flaw echoes from the rough crack in time and frequency domain. (**a**) Multiple repetitions in time domain; (**b**) multiple repetitions in frequency domain; (**c**) statistical results in time domain; (**d**) statistical results in frequency domain. The width of the notch was 0.02 mm.

Figure 9 shows the statistical findings of 100 groups of different flaw echoes in the time and frequency domains for a rough crack. By use of the Hilbert transform, the signal envelope is obtained from the time-domain waveform signal, which is then normalized by the maximum front-wall echo. Meanwhile, the corresponding frequency-domain signal for the flaw echo is acquired in 14 to 16 µs through fast Fourier transform. Figure 9a shows that the randomness and complexity of flaw echoes are exceptionally high. Figure 9b shows that the peak frequencies occur randomly in the range of 2.5 ± 1.25 MHz. However, there may have been nonlinear flaw scattering processes, such as the generation of subharmonics. In Figure 9c,d, the dark red line represents the ensemble average, whereas the red region represents the range of one standard deviation. The amplitude of the ensemble average of flaw echoes from the rough crack drops by −7.13 dB when compared with the flaw waves of the same-sized smooth notch (0.02 mm width and 3 mm depth). The flaw echo of rough flaws is less than that of the standard reflector, which is in accordance with previous experience and experimental results. In addition, the variation in the rough defect wave at this frequency is more noticeable because the center frequency of the transducer is 2.5 MHz.

**Figure 10:**
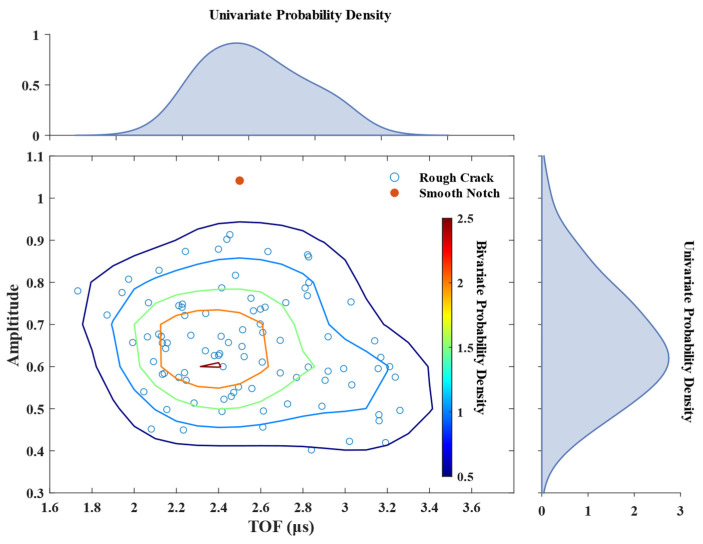
Scatter plot of the amplitude and TOF.

The joint distribution of the amplitude and TOF of the peak of flaw echoes are shown in Figure 10. The maximum and minimum values of the normalized amplitude are 0.91 and 0.40, and their difference is greater than double. However, the maximum amplitude of the rough crack is still less than that of the smooth notch. The maximum and minimum values of the TOF are 3.48 and 1.73 µs, and the gap between them is about double. The peak of the TOF distribution is close to the TOF of the smooth notch. These results show that after multi-scale distortion, the flaw scattering of rough cracks becomes unexpected. This makes it challenging to locate and quantify them.

**Figure 11:**
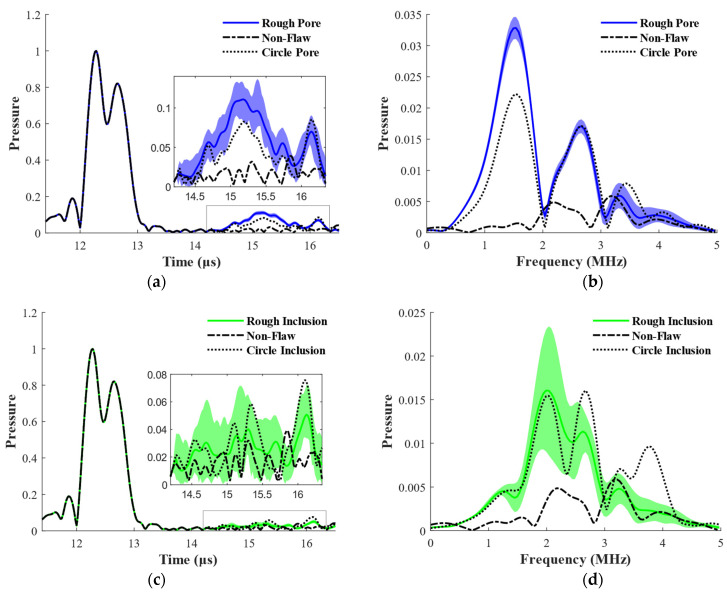
Statistical results for the rough pore and rough inclusion. (**a**) Rough pore, time domain; (**b**) rough pore, frequency domain; (**c**) rough inclusion, time domain; (**d**) rough inclusion, frequency domain.

Figure 11 shows the statistical results for the rough pore and rough inclusion. From the range of one standard deviation, the fluctuations of rough pore and rough inclusion are 1.22 dB and 1.93 dB, respectively. This may happen because their equivalent sizes are all smaller than the wavelength of the transverse wave, with the pore size being 0.08 times the wavelength and the inclusion size being 0.81 times the wavelength, whereas the wavelength of the transverse wave at 2.5 MHz is 1.23 mm in the nickel-based bar. Therefore, a 2.5 MHz transducer will be unsuitable if small flaws must be detected during the actual inspection. We anticipate that the roughness-induced fluctuation will increase when increasing the inspection frequency or shortening the wavelength of the transverse wave. Additionally, it is interesting to observe that after multi-scale distortion, both the flaw echoes of rough pore and rough inclusion are higher than those of the equivalent-sized standard reflector. More specifically, their leading-edge responses are noticeably stronger than those caused by circular flaws. This may be caused by the macroscopic rough geometry of the distorted flaw. This requires a more detailed study in the future.

## 4. Conclusions

In this work, the image distortion technique from the optical imaging field is first introduced for the generation of a rough flaw for the FE simulation of ultrasonic flaw scattering. The image distortion technique was briefly reviewed, and a multi-scale ripple distortion method for operating the seed of the OM image of a real flaw was developed, which resulted in the generation of rough flaws that have a feeling of realism. The proposed method has demonstrated its superiority in generating rough cracks with secondary cracks, rough pores, and rough inclusions. We then selected the ultrasonic inspection of surface or sub-surface flaws in nickel-based bars, using a mode-converted transverse wave as an example. To perform the 2D FE simulations, the rough flaw was positioned on the surface or sub-surface of the bar. The numerical results show that the macroscopic rough geometry and the microscopic rough surface of the flaw may be to blame for the randomness of amplitude and TOF in the flaw echoes. Furthermore, our simulations revealed that it would be extremely challenging to quantify the size of a rough crack with secondary cracks.

Finally, it should be noted that the present work has observed several limitations. First, the parameter combination settings of ripple distortion that are presented here are for illustrative purposes only. The use of uniform distribution has not been experimentally verified and is not mandatory. Future industrial applications require research into the explainable parameter combination of ripple distortion, which will be useful for controlling the correlation length and the arithmetic mean roughness. Second, limited by computing resources, our simulation frequency is lower than the frequency of real inspection. Third, only 2D simulations are performed, but 3D simulations for rough crack, rough pore, and rough inclusion are necessary to compare the experiments. Finally, experimental verification for rough flaw inspection is unavailable. These limitations also provide us insight into where to focus our efforts in the future. In addition, the practical applications may extend beyond the field of ultrasonic nondestructive evaluation to include medical ultrasound diagnosis, quantitative seismology, and seabed sediment acoustics.

## Figures and Tables

**Table 1:** Properties of reference nickel-based alloy and non-metallic inclusion.

Materials	Elastic Modulus (GPa)	Density (g/cm^3^)	Poisson Ratio
Nickel-based alloy [21]	200	8.24	0.29
Al_2_O_3_-MgO-TiN [22]	255	6.58	0.25

## Data Availability

Due to the nature of this research, since the participants of this study did not agree for their data to be shared publicly, the supporting data are not available.
